# p62 Promotes the Mitochondrial Localization of p53 through Its UBA Domain and Participates in Regulating the Sensitivity of Ovarian Cancer Cells to Cisplatin

**DOI:** 10.3390/ijms23063290

**Published:** 2022-03-18

**Authors:** Qinghuan Kong, Xiaoyu Yan, Meiyu Cheng, Xin Jiang, Long Xu, Luyan Shen, Huimei Yu, Liankun Sun

**Affiliations:** 1 Department of Pathophysiology, College of Basic Medical Sciences, Jilin University, Changchun 130021, China; akashihime@163.com (Q.K.); yanxy@jlu.edu.cn (X.Y.); mycheng19@mails.edu.cn (M.C.); xulong20@mails.jlu.edu.cn (L.X.); shenly@jlu.edu.cn (L.S.); 2 Department of Biochemistry, College of Basic Medical Sciences, Jilin University, Changchun 130021, China; xinjiang@jlu.edu.cn

**Keywords:** p53, p62/SQSTM1, ovarian cancer, drug resistance, mitochondria

## Abstract

Chemotherapeutic drug-induced p53-dependent crosstalk among tumor cells affects the sensitivity of tumor cells to chemotherapeutic drugs, contributing to chemoresistance. Therefore, pharmacological targeting of p53 may contribute to overcoming drug resistance. The localization of p53 is closely related to its function. Thus, we assessed the effect of p62 on the coordination of p53 mitochondrial localization under chemotherapeutic drug treatment in ovarian cancer cells. We found that the combined use of the proteasome inhibitor epoxomicin and cisplatin led to the accumulation of p53 and sequestosome1(p62) in the mitochondria, downregulated mitochondrial DNA (mtDNA) transcription, inhibited mitochondrial functions, and ultimately promoted apoptosis by enhancing cisplatin sensitivity in ovarian cancer cells. Moreover, the ubiquitin-associated (UBA) domain of p62 was involved in regulating the mitochondrial localization of p53. Our findings suggest that the interaction between p62 and p53 may be a mechanism that determines the fate of tumor cells. In conclusion, p62 coordinated the mitochondrial localization of p53 through its UBA domain, inhibited mtDNA transcription, downregulated mitochondrial function, and promoted ovarian cancer cell death. Our study demonstrates the important role of p53 localization in tumor cell survival and apoptosis, and provides new insights into understanding the anti-tumor mechanism of targeting the ubiquitin–proteasome system in tumor cells.

## 1. Introduction

Ovarian cancer is the most lethal gynecological malignancy [1]. Surgery and platinum-based chemotherapy are the main treatments for ovarian cancer. However, ovarian cancer cell resistance to platinum-based drugs is the main cause of death in ovarian cancer patients [2,3]. Studies have shown that the proteasome inhibitor MLN9708 induced breast cancer cell sensitivity to doxorubicin and that the proteasome inhibitor oprozomib increased the sensitivity of cervical cancer to cisplatin [4,5]. These findings suggest that targeting the ubiquitin–proteasome system (UPS) of tumor cells to interfere with cancer cell proteostasis will increase the sensitivity of cancer cells to chemotherapeutic drugs such as cisplatin. The targets of chemotherapeutic drugs are complex, which results in diverse changes in tumor cells and the formation of interaction networks. To explore the mechanism of these changes, we investigated the role of sequestosome 1/p62 (SQSTM1/p62, hereafter referred to as p62) in ovarian cancer cells and found that p62 formed a “death platform” with autophagosome membranes to recruit Caspase 8 and regulate its activation in ovarian cancer cells [6]. It has been suggested that biomolecular condensates are formed when cells respond to stress, indicating that proteins and RNAs in tumor cells may need to interact with other molecules to exert their function [7]. These findings suggest that exploring the interaction between various molecules and proteins may provide insights into the mechanism underlying the anti-tumor effects of UPS inhibition.

p53 performs its tumor suppressor function by inducing tumor cell apoptosis and cell cycle arrest [8]. In addition, p53 also plays an important role in the regulation of cellular metabolism, hypoxia, DNA repair, genome stability, aging, and metabolic homeostasis [9,10,11]. Loss of p53 function allows abnormal cell proliferation and is closely associated with carcinogenesis. The stability and anti-tumor properties of p53 are closely related to the UPS which strictly regulates p53 degradation [12]. Various drugs targeting the p53 ubiquitination degradation pathway have been developed for cancer treatment [13,14]. Furthermore, the function of p53 is closely related to its subcellular localization. Tsademir et al. found that p53 localized in the cytoplasm of the human colon cancer cell line HCT116 promoted autophagy, while p53 localized in the nucleus inhibited autophagy [15]. p53 localized in the nucleus usually acts as a transcription factor regulating the transcription of proapoptotic genes, such as *Puma*, *Noxa*, and *Bax*, indirectly promoting apoptosis. Furthermore, Chipuk et al. found that p53 localized in mitochondria directly activated Bax to mediate mitochondrial membrane permeabilization and apoptosis [16,17]. These findings suggest that inhibition of the proteasome pathway may affect the subcellular localization of p53. This could provide clues for developing new targets to for cancer therapy.

Recent evidence suggests that the mitochondrial unfolded protein response (UPR^mt^) is a retrograde signaling pathway from the mitochondria to the nucleus, which attenuates mitochondrial stress, adapts tumor cells to various forms of mitochondrial dysfunction, maintains mitochondrial protein stability, and prevents mitochondria-induced cell death in vivo [18,19]. Therefore, it is considered to be a promising target for tumor therapy. The UPR^mt^ can be activated upon loss of oxidative phosphorylation (OXPHOS) function or unfolded protein accumulation in the mitochondria. A study in *Caenorhabditis elegans* by Nargund et al. showed that the UPR^mt^ is a transcriptional response mediated by the transcription factor activating transcription factor associated with stress-1(ATFS-1) [20], activating transcription factor 5 (ATF5) in *mammals* [21],which promotes cell survival by enhancing the recovery and regeneration of defective mitochondria. Furthermore, ATF5 promotes cancer cell survival by inducing the transcription of the pro-apoptotic gene Mcl-1 [22]. Moreover, the UPR^mt^ activates the transcription of genes that assemble OXPHOS complexes to promote cell survival [23]. Studies have shown that p53 regulates mitochondrial proteins involved in mitochondrial metabolism and respiration. For example, Sahin et al. found that telomere damage induces p53-mediated transcriptional repression of the expression of *PGC-1α* and *PGC-1β*, thereby inhibiting the transcription of *PGC-1α* transcriptional targets including transcription factor A, mitochondrial (*TFAM)* in various mouse tissues [24]. The findings of a study that explored the link between p53 and the UPR^mt^ following denervation-induced muscle disuse of skeletal muscle, suggested that p53 affected mitochondrial protein homeostasis by mediating a series of mitochondrial quality control processes including the UPR^mt^ [25]. Therefore, exploring the link between p53 and the UPR^mt^ in tumors may provide evidence supporting the inhibition of the proteasome pathway to increase chemotherapeutic drug sensitivity [26,27].

p62 is well known as the most typical autophagy receptor participating in the degradation of cellular proteins [28]. Numerous studies have found that p62 acts as a “double-edged sword” and plays an important role in tumors and regulates cell fate determination. Studies of knockout mice have confirmed that p62 deletion significantly delayed tumorigenesis and development, suggesting that primary p62 might be involved in the regulation of pro-survival signals [29]. However, Zhang et al. found that accumulation of p62 induced by the protease inhibitor MG132 increased HAMLET-induced apoptosis in U87MG cells, suggesting that the accumulation of p62 induced by the proteasome inhibitor was involved in the regulation of pro-death signaling [30]. As a signal hub, the function of p62 depends on its multiple domains. For example, through the LC3-interacting region (LIR) domain, p62 interacts with microtubule-associated protein light chain 3 (LC3), which is required for autophagosome biosynthesis, enabling p62 to play a key role in autophagy [31]. p62 interacts with polyubiquitinated proteins through its C-terminal ubiquitin-associated (UBA) domain, which enables p62 to shuttle ubiquitinated proteins for proteasomal degradation [32]. As a shuttle protein, p62 also functions as a transporter. p62 contributes to the recruitment of proteasomes to nuclear aggregates of ataxin-1 and the subsequent degradation of ataxin-1, which also recruits nuclear polyubiquitinated proteins to the promyelocytic leukemia(PML) bodies [33]. Noguchi et al. found that p62-based aggresome-like induced structures (ALIS) mediates cefotaxime-induced parthanatos in human fibrosarcoma by promoting the nuclear translocation of AIF, suggesting that p62 regulates cell death through its transport function [34]. Although few reports investigated the regulatory relationship between p53 and p62 in tumors, some studies have shown that p53 stability was attenuated in p62-deficient HCT116 cells after prolonged mitosis [35]. Furthermore, induction of p62 by MLN4924 was dependent on p53 in vascular smooth muscle cells and mediated MLN4924-induced apoptosis [36]. Thus, we speculated that accumulation of p62 induced by the proteasome pathway may be involved in the transport of p53 to determine cell fate.

In this study, we hypothesized that p62 played a role in the subcellular localization of p53 in ovarian cancer cells. We found that the combined use of epoxomicin (epox) with cisplatin increased mitochondrial localization of p53 through the UBA domain of p62, thereby downregulating mitochondrial functions, affecting the transcription of important subunits of the mitochondrial OXPHOS system and the UPR^mt^, and mediating cell death. These findings suggest that targeting the proteasome pathway may inhibit tumor cell proliferation.

## 2. Results

### 2.1. High p53 Expression Was Associated with Cisplatin Sensitivity in Ovarian Cancer Cells

**Figure 1:**
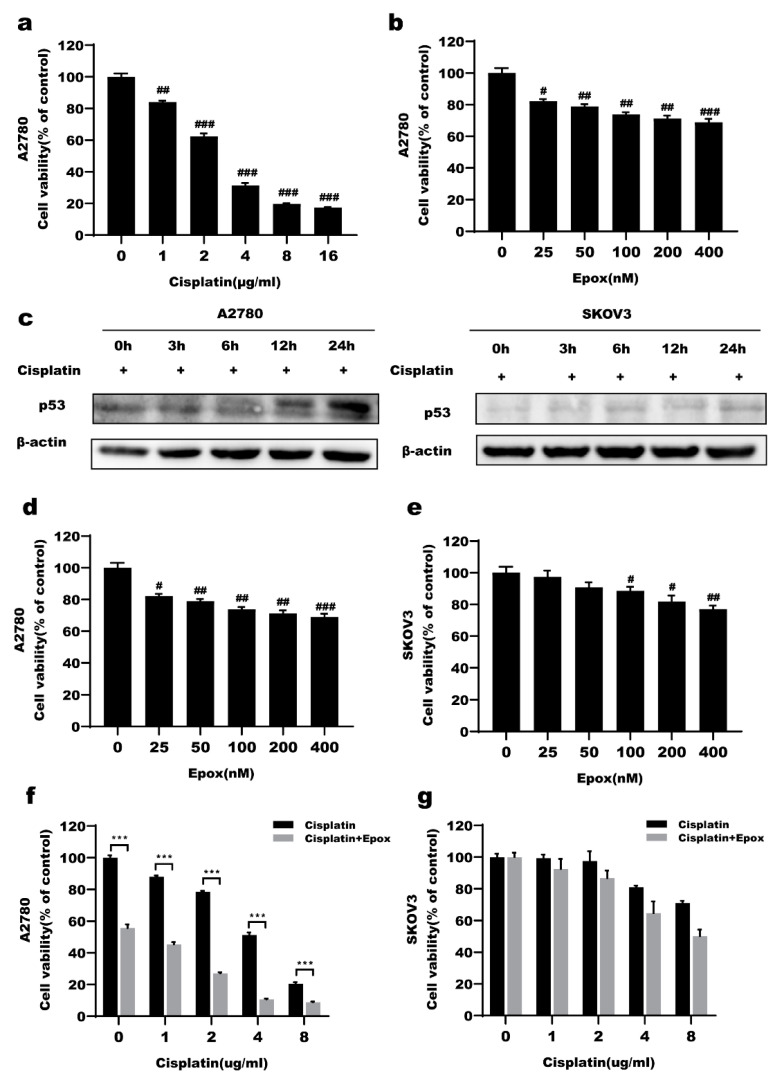
High expression of p53 is related to cisplatin sensitivity of ovarian cancer cells. (**a**,**b**) Cells were treated with cisplatin for 24 h. The cell viability of A2780 cells and SKOV3 cells were detected by MTT assay. (**c**) p53 protein levels were detected by western blot after treatment with cisplatin (2 μg/mL). (**d**,**e**) Cells were treated with epoxomicin(epox) for 24 h. The cell viability of A2780 cells and SKOV3 cells detected by MTT assay. (**f**,**g**) Cell viability of A2780 cells and SKOV3 cells were detected by MTT after treated with cisplatin or combined with epox (100 nM) for 24 h. # *p* < 0.05 vs. con, ## *p* < 0.01 vs. con, ### *p* < 0.001 vs. con., *** *p* < 0.001 vs. Cisplatin.

A2780 and SKOV3 cells were treated with cisplatin(CDDP) for 24 h and cell viability was evaluated using MTT assays. We found that A2780 cells were more sensitive to cisplatin than SKOV3 cells (Figure 1a,b). Under cisplatin treatment at a dose of 2 µg/mL, the protein level of p53 in A2780 cells was increased in a time-dependent manner, but the p53 protein level in SKOV3 cells did not significantly change, indicating that p53 might be related to ovarian cancer cell cisplatin sensitivity (Figure 1c). To verify the role of p53 in ovarian cancer cisplatin sensitivity, the proteasome inhibitor epox was used to inhibit p53 protein degradation. Cells were treated with epox for 24 h and MTT assays showed that A2780 cells were more sensitive to epox than SKOV3 cells (Figure 1d,e). After combined treatment with CDDP and epox, the viability of A2780 cells exposed to cisplatin significantly decreased, while that of SKOV3 did not significantly change (Figure 1f,g). Therefore, A2780 cells were used in the following experiments with a cisplatin dose of 2 μg/mL and epox dose of 100 nM.

### 2.2. Epox-Induced Proteasome Inhibition Led to Mitochondrial Dysfunction and UPR^mt^ in Ovarian Cancer Cells

**Figure 2:**
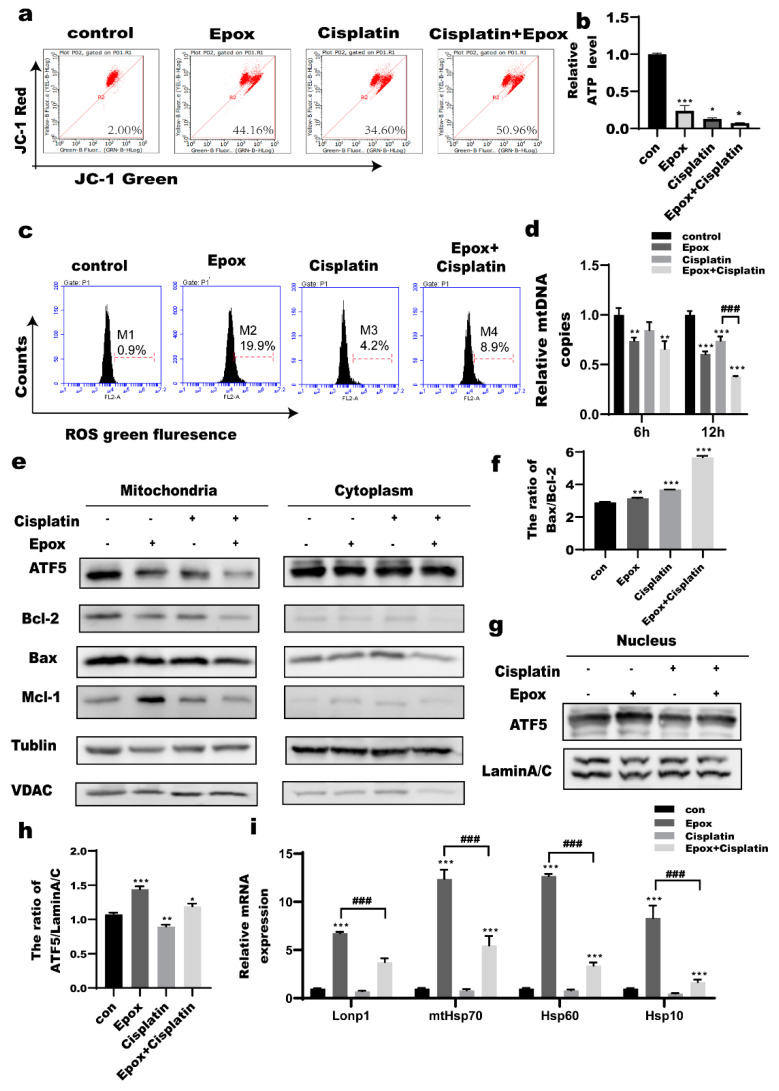
Epox−induced proteasome inhibition leads to mitochondrial dysfunction and UPR^mt^ in ovarian cancer cells. (**a**) A2780 cells were treated with epox (100 nM) or CDDP (2 μg/mL) or both for 24 h. Mitochondrial membrane potential detected by flow cytometry. (**b**) A2780 cells were treated with epox (100 nM) or CDDP (2 μg/mL) or both for 12 h. ATP production was measured based on total protein content amount. (**c**) A2780 cells were treated with epox (100 nM) or CDDP (2 μg/mL) or both for 12 h. Mitochondrial ROS generation was detected by flow cytometry detection. (**d**) A2780 cells were treated with epox (100 nM) or CDDP (2 μg/mL) or both for 12 h. Quantitative RT-PCR validation of mitochondrial DNA (mtDNA) copy number. (**e**) A2780 cells were treated with epox (100 nM) or CDDP (2 μg/mL) or both for 12 h. Protein levels of activating transcription factor 5(ATF5), apoptosis-related proteins in mitochondria were detected by western blot. (**f**) Quantitation of (B cell lymphoma-2)Bax/(B cell lymphoma-2)Bcl-2. (**g**,**h**) ATF5 nuclear protein levels detected by western blot (12 h). (**i**)Quantitative RT-PCR detection of mitochondrial unfolded protein response (UPR^mt^) related mRNAs level. * *p* < 0.05 vs. con, ** *p* < 0.01 vs. con, *** *p* < 0.001 vs. con, ### *p* < 0.001 vs. Epox + Cisplatin.

A2780 cells were treated with epox (100 nM) or cisplatin (2 μg/mL) or both for 0, 6, 12, and 24 h to assess mitochondrial functions. The mitochondrial membrane potential at 24 h and ATP production at 12 h decreased in the epox, CDDP, and combined CDDP and epox groups. These changes were the most obvious in the combined CDDP and epox group (Figure 2a,b). Production of mitochondrial reactive oxygen species (ROS) at 12 h increased in the combined CDDP and epox group compared with that of the CDDP group (Figure 2c). As mitochondrial functions are closely related to mitochondrial DNA (mtDNA) integrity that partly depends on the mtDNA copy number [37,38]. Therefore, we measured the mtDNA copy number. At 12 h, the mtDNA copy number in the combined CDDP and epox group decreased significantly compared with that in the CDDP group (Figure 2d). The UPR^mt^ protects cells from a wide range of mitochondrial stresses that include oxidative phosphorylation (OXPHOS) dysfunction, disturbance of protein import due to mitochondrial protein misfolding, ATP depletion, and dissipation of the mitochondrial inner membrane potential [20]. Mitochondria were isolated to detect the levels of common markers associated with apoptosis and those of ATF5 after treatment with epox (100 nM) or cisplatin (2 μg/mL) or both for 12 h. The levels of mitochondrial ATF5 decreased in the epox, CDDP, and combined CDDP and epox groups. The most obvious decease in the mitochondrial ATF5 levels was observed in the combined CDDP and epox group. The levels of the anti-apoptotic proteins B cell lymphoma-2 (Bcl-2) and myeloid cell leukemia-1(MCL-1) decreased significantly and the Bcl-2-associated X (Bax)/Bcl-2 ratio increased in the combined CDDP and epox group compared with those in the epox and CDDP groups (Figure 2e,f). However, in the epox and combined epox and CDDP groups, the levels of nuclear ATF5 were slightly increased and the mRNA levels of *LONP1*, *mtHSP70*, *HSP60*, and *HSP10*, which play a protective role as the downstream of ATF5, were increased, but their levels in the combined CDDP and epox group were lower compared with those in the epox group (Figure 2g–i). These results suggested that the cells initiated the UPR^mt^ as a protection mechanism against stress, but ultimately this protection did not allow the cells to survive. 

### 2.3. Inhibition of the Proteasome Pathway Affected the Expression of Mitochondrial Respiratory Chain Subunits in Ovarian Cancer Cells

**Figure 3:**
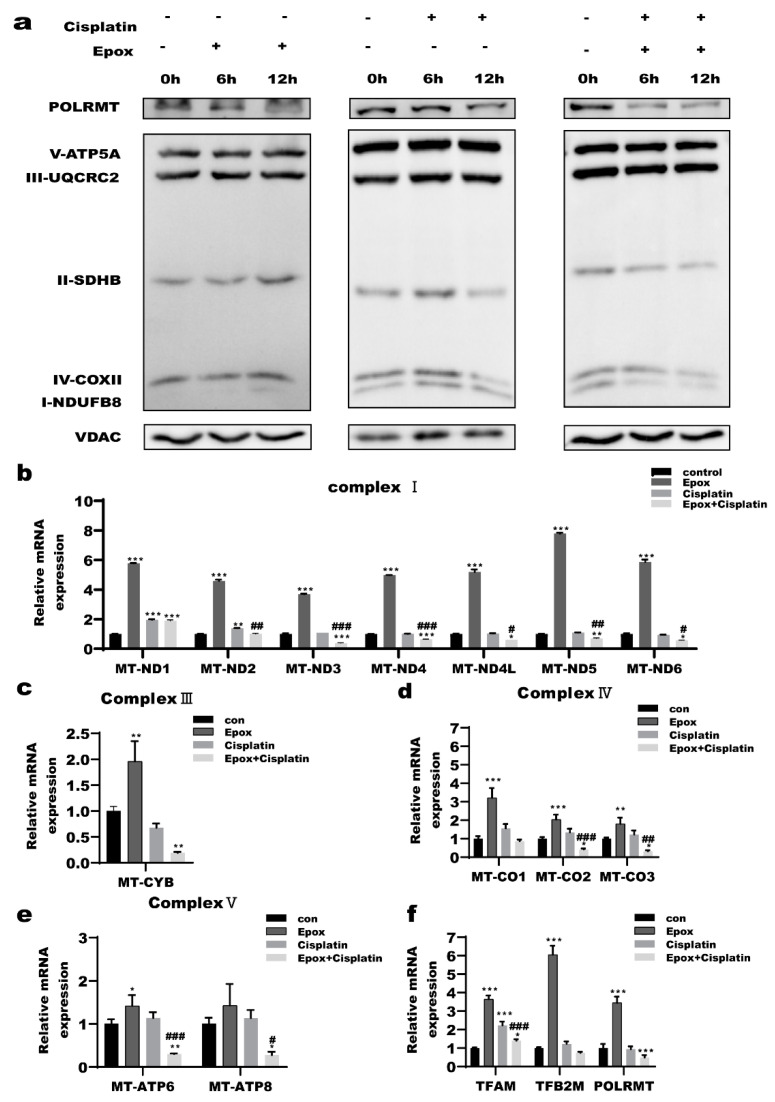
Inhibition of the proteasome pathway affected the expression of respiratory chain subunits in ovarian cancer cells. (**a**) A2780 cells were treat with cisplatin (2 μg/mL) or epox (100 nM) or both for 3, 6, 12 h. Western blot analysis of mitochondrial subunits and mitochondrial RNA polymerase (POLRMT) levels. (**b**–**e**) A2780 cells were treat with cisplatin (2 μg/mL) or epox (100 nM) or both for 12 h. Quantitative RT-PCR detection of mtDNA-encoded respiratory chain subunits and (**f**) mitochondrial DNA transcription complex mRNAs level. * *p* < 0.05 vs. con, ** *p* < 0.01 vs. con, *** *p* < 0.001 vs. con, # *p* < 0.05 vs. Cisplatin, ## *p* < 0.01 vs. Cisplatin, ### *p* < 0.001 vs. Cisplatin.

**Figure 4:**
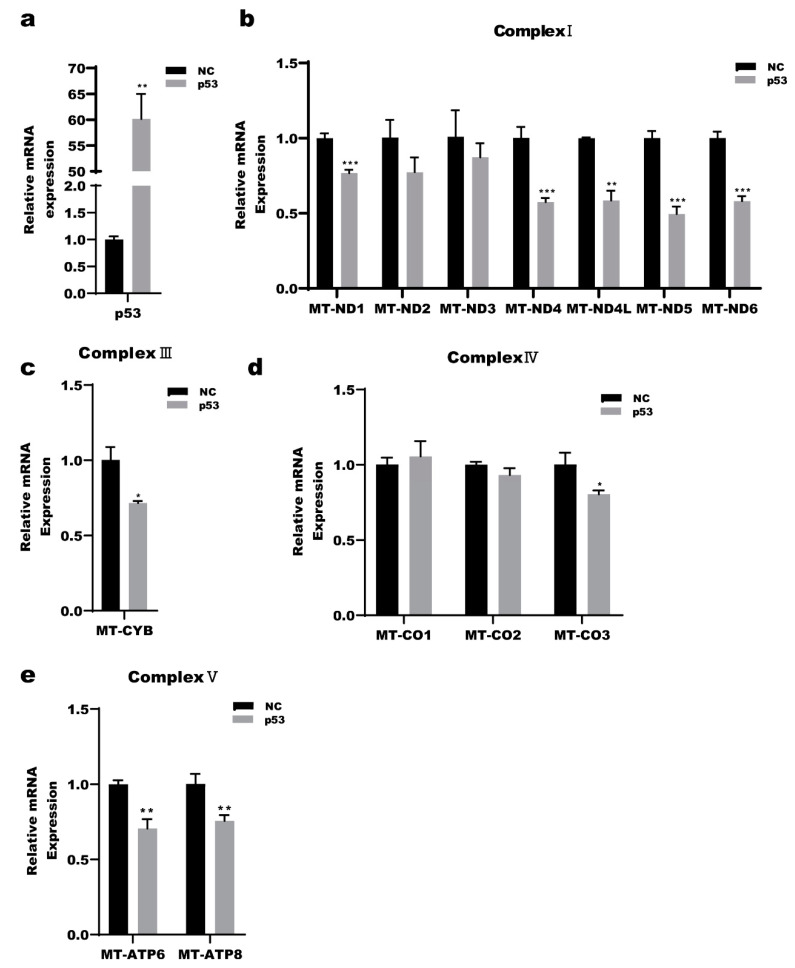
p53 is involved in the transcription respiratory chain subunits encoded by mtDNA. Wild-type p53 were transfected into A2780 cells for 24 h and total RNA were extracted. (**a**) Quantitative RT-PCR detection of the transfection efficiency of wild-type p53 transfected into A2780 cells. (**b**–**e**) Quantitative RT-PCR detection of mRNAs level of respiratory chain subunits encoded by mtDNA. * *p* < 0.05 vs. NC, ** *p* < 0.01 vs. NC, *** *p* < 0.001 vs. NC.

The mitochondrial OXPHOS system consists of 90 proteins whose biogenesis is under dual genetic control and requires the coordinated expression of nuclear and mitochondrial genes [39]. The 13 core subunits of the respiratory chain complex are encoded by mtDNA. Human mtDNA encodes seven subunits of mitochondrial respiratory chain complex I (ND1, ND2, ND3, ND4, ND4L, ND5, and ND6), one subunit of complex III (cytochrome b), and three subunits of complex IV bases (COXI, COXII and COXIII), two subunits of complex V (ATPases 6 and 8). mtDNA transcription is completely dependent on nuclear DNA-encoded factors [40]. The human mitochondrial DNA is transcribed by mitochondrial RNA polymerase (POLRMT) and requires two initiation factors, namely, the mitochondrial transcription factors A (TFAM) and B2 (TFB2M) to form a transcription complex [41,42]. A2780 cells were treated with epox (100 nM) or cisplatin (2 μg/mL) or both for 0, 6, 12 h. We examined the protein levels of nuclear DNA-encoded mitochondrial subunits, the transcriptional complex and the mRNA levels of the 13 mtDNA-encoded respiratory chain subunits encoded by mtDNA and the mitochondrial transcription complex. The results showed that the POLRMT levels were significantly decreased in the combined CDDP and epox group at 6 h and 12 h compared with those in the epox and CDDP groups. In the epox group, the succinate dehydrogenase complex iron sulfur subunit B(SDHB), cytochrome c oxidase subunit II(COXII), and NADH:ubiquinone oxidoreductase subunit B8(NDUFB8) levels were slightly increased at 12 h, whereas those in the CDDP group did not significantly change at 6 h, but decreased at 12 h. In the combined CDDP and epox group, the levels of SDHB and NDFUBB were decreased in a time-dependent manner, while those of COXII were increased at 6 h and then decreased at 12 h (Figure 3a). The mRNA expression levels of most of the 13 respiratory chain subunits encoded by mtDNA were significantly decreased in the combined CDDP and epox group, except for those of *MT-ND2* and *MT-CO1* (Figure 3b–e). Furthermore, the expression levels of *TFAM* and *POLRMT* were decreased in the combined CDDP and epox group (Figure 3f). To verify the regulatory role of p53 in mitochondrial DNA transcription, wild-type p53 was transfected into A2780 cells and the mRNA levels of the 13 respiratory chain subunits were examined (Figure 4a). The results showed that the mRNA levels of most subunits were decreased in the combined CDDP and epox group, except those for *MT-ND2*, *MT-ND3*, *MT-CO1*, and *MT*-CO2 (Figure 4b–e). These results indicated that p53 might be involved in the mtDNA-regulated transcription of respiratory chain subunits.

### 2.4. Inhibition of the Proteasome Pathway Promoted Mitochondrial Localization of p53 in Ovarian Cancer Cells

**Figure 5:**
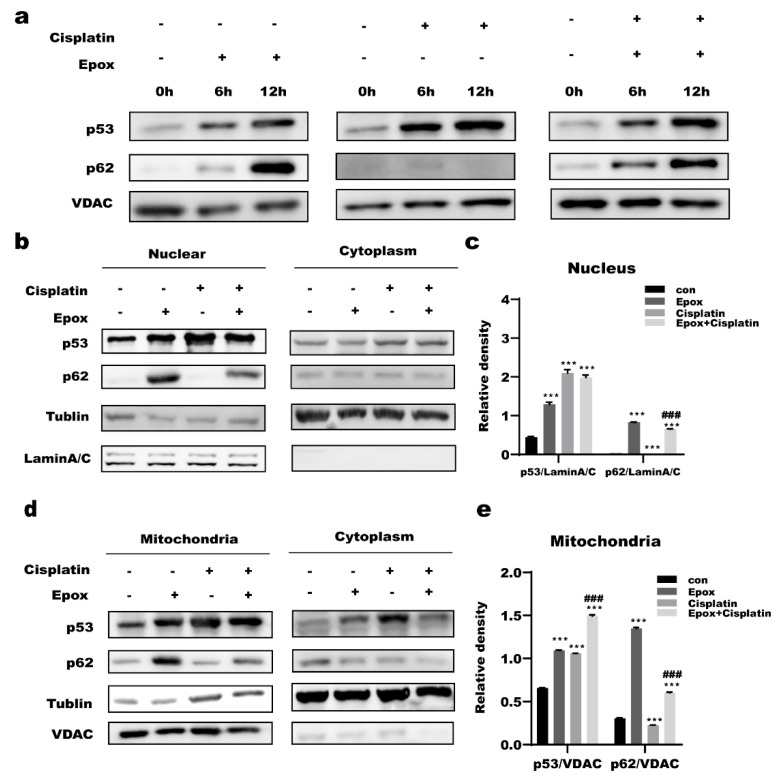
Inhibition of proteasome pathway promoted mitochondrial localization of p53 and p62 in ovarian cancer cells. (**a**) A2780 cells were treat with cisplatin (2 μg/mL) or epox (100 nM) or both for 0,6,12 h. Sequestosome 1(p62) and p53 protein levels in mitochondria were measured by western blot. (**b,c**) A2780 cells were treat with cisplatin (2 μg/mL) or epox (100 nM) or both for 12 h. p62 and p53 protein levels in mitochondria were measured by western blot. (**d**,**e**) A2780 cells were treat with cisplatin (2 μg/mL) or epox (100 nM) or both for 12 h.p62 and p53 protein levels in nucleus were measured by western blot. *** *p* < 0.001 vs. con; ### *p* < 0.001 vs. Cisplatin.

**Figure 6:**
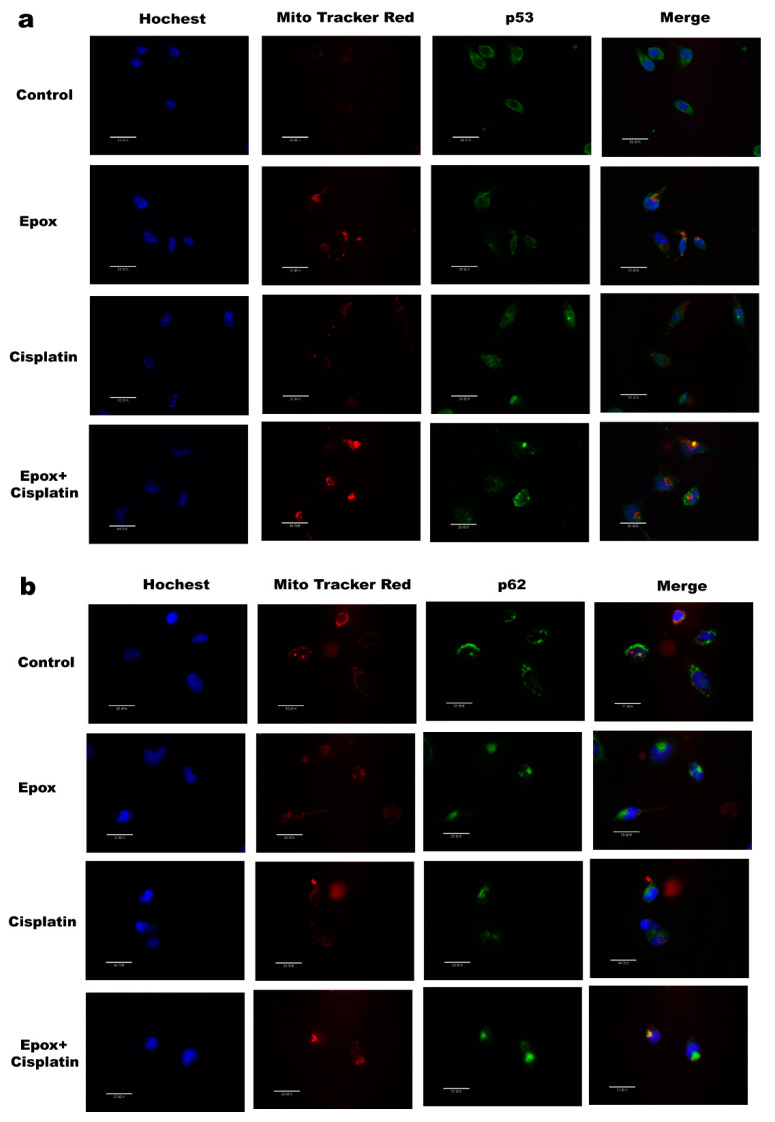
Inhibition of proteasome pathway promoted mitochondrial localization of p53 and p62 in ovarian cancer cells. A2780 cells were treat with cisplatin (2 μg/mL) or epox (100 nM) or both for 12 h. (**a**,**b**) Immunofluorescence staining for p53, p62 and mitochondria in A2780 cells (bar = 30 μm).

The function of p53 is closely related to its subcellular localization. A2780 cells were treated with epox (100 nM) or cisplatin (2 μg/mL) or both for 0,6,12 h and the mitochondria and nucleus were isolated to determine the subcellular localization of p53. We found that the levels of p53 and p62 increased in a time-dependent manner in the epox and the combined CDDP and epox groups, while only the levels of p53 showed a time-dependent increase in the CDDP group in the mitochondria (Figure 5a). Compared with the CDDP group, the levels of nuclear p53 in the combined CDDP and epox group were slightly decreased, and those of p62 were increased, while those of p53 significantly increased in the mitochondria (Figure 5b–e).Immunofluorescence staining showed that the combination use of epox and CDDP promotedp53 and p62 accumulation in the mitochondria in A2780 cells (Figure 6a,b).

### 2.5. UBA Domain Deletion in p62 Inhibited Mitochondrial Localization of p53

**Figure 7:**
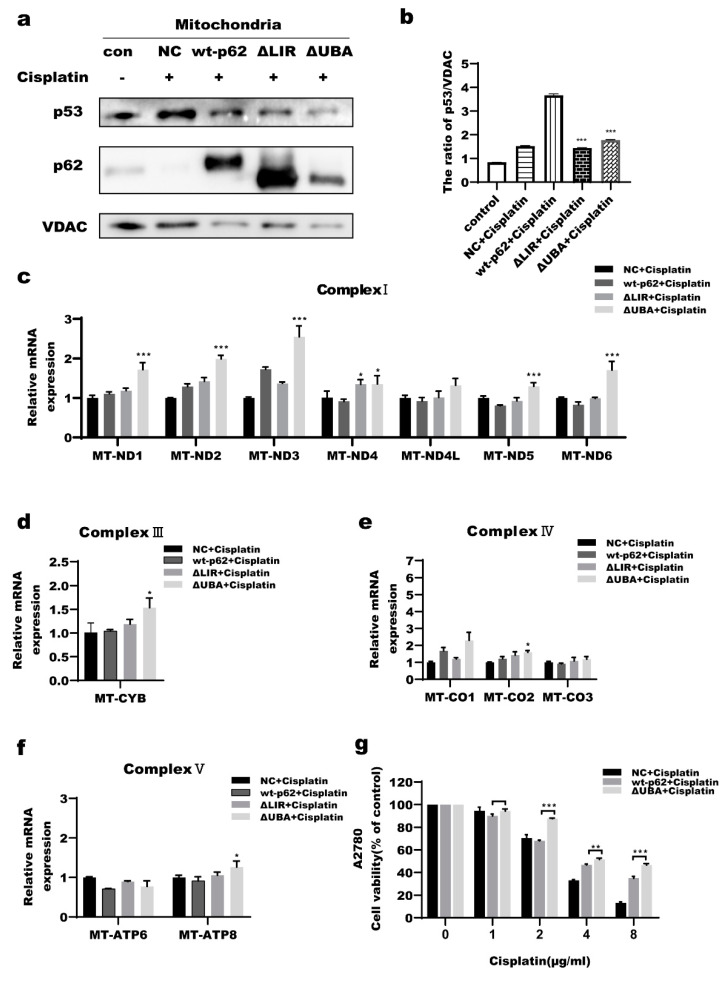
Deleting the ubiquitin-associated (UBA) domain of p62 inhibited mitochondrial localization of p53 in A2780 cells. (**a**,**b**) A2780 cells were treat with cisplatin (2 μg/mL) for 12 h after transfection with different types of p62 for 24 h. Western blot analysis of p53 protein levels in the mitochondria. (**c**−**f**) A2780 cells were treat with cisplatin (2 μg/mL) for 12 h after transfection with different types of p62 for 24 h. Quantitative RT-PCR detection of mRNAs level of encoding respiratory chain subunits encoded by mtDNA. (**g**) A2780 cells were treated with CDDP for 24 h after transfection for 24 h. Cell viability was analyzed by MTT after cisplatin treatment for 24 h. * *p* < 0.05 vs. wt-p62, ** *p* < 0.01 vs. wt-p62, *** *p* < 0.001 vs. wt-p62.

p62 is a scaffold protein with multiple functional motifs which plays important roles in both the UPS and autophagy [43]. Wild-type p62 (wt-p62), a UBA domain truncation mutant (ΔUBA) of p62, and an LIR domain truncation mutant (ΔLIR) of p62 were transfected into A2780 cells. After treatment with CDDP for 12 h at a dose of 2 μg/mL, mitochondria were isolated to detect the mitochondrial localization of p53. The levels of mitochondrial p53 were decreased in the ΔUBA and ΔLIR groups compared with those of the wt-p62 group (Figure 7a,b). Total mRNA was extracted to examine the effect of the different types of p62 on mtDNA transcription under the same treatments. Compared with the wt-p62 group, the mRNA levels of the mtDNA-encoded respiratory chain subunits were significantly increased in the ΔUBA group (except for *MT-CO1* and *MT-CO3*), but were not significantly altered in the ΔLIR group (Figure 7c–f). Cell viability was examined to verify the effect of the UBA domain on cell fate. After treatment with CDDP for 24 h, cell viability was significantly higher in the ΔUBA group compared with that of the wt-p62 group (Figure 7g). These data suggest that p62 participated in the mitochondrial translocation of p53 through its UBA domain, thereby regulating the transcription of mtDNA-regulated respiratory chain subunits, which made the cells less sensitive to cisplatin and enhanced cell survival.

## 3. Discussion

Chemotherapy resistance remains a major obstacle that limits ovarian cancer treatment [44]. The UPS plays a major role in maintaining protein homeostasis [45]. Therefore, the role of the proteasome pathway in the occurrence and development of tumors has become a research hotspot. Recent studies have shown that the UPS participates in the regulation of cell proliferation, apoptosis, and other pathways by regulating nuclear transcription factors such as the nuclear factor NF-kappaB (NF-κB) and p53 [46]. It also mediates the proteasomal degradation of mitochondrial proteins, such as MCL-1, to regulate the mitochondria-dependent apoptosis pathway [47], which suggests that further research on the role of the UPS in nuclear–mitochondrial interaction signaling may provide a basis for the development of UPS inhibitors to increase the sensitivity of traditional anti-cancer drugs.

In this study, we found that A2780 cells are more sensitive to cisplatin than SKOV3 cells (Figure 1a,b). Under treatment with CDDP, the p53 protein level in A2780 cells increased in a time-dependent manner (Figure 1c). The proteasome inhibitor epox could significantly increase the sensitivity of A2780 cells to cisplatin but there was no significant change in the cisplatin sensitivity of SKOV3 cells (Figure 1f,g). Furthermore, when epox was applied in combination with CDDP for 12 h, mitochondrial p53 expression was increased, while nuclear p53 expression was slightly decreased (Figure 5a–d and Figure 6a,b). These results suggest that the accumulation of mitochondrial p53 caused by the inhibition of the proteasome pathway might be related to ovarian cancer cell CDDP sensitivity.

Mitochondria regulate cellular energy metabolism and free radical production and are the most representative organelles that perform programmed cell death [48]. Tsakiri et al. demonstrated that a decrease in proteasome functions could lead to severe defects in mitochondrial functions [49]. We found that when CDDP treatment was combined with epox in A2780 cells, mitochondrial membrane potential decreased, ATP production decreased, mitochondrial ROS production increased, and the mtDNA copy number decreased, the protein levels of the anti-apoptotic proteins Bcl-2 and Mcl-1 in the mitochondria decreased, and the Bax/Bcl-2 ratio increased, (Figure 2a–e). These data indicated that the accumulation of p53 in mitochondria caused by the combined treatment with epox and CDDP might be related to the mitochondria-dependent apoptosis pathway.

Under mitochondrial stress, the UPR^mt^ upregulates the expression of molecular chaperones, such as HSP10, HSP60, mtHSP70, and Lonp1, and genes that encode OXPHOS complexes to protect the respiratory chain and inhibit mitochondrial pathway-induced apoptosis [23]. In this study, we found that the mRNA levels of *HSP10*, *HSP60*, *mtHSP70*, and *Lonp1* were increased in the epox group, but their mRNA levels were significantly decreased when CDDP treatment was combined with epox((Figure 2h). In addition, mitochondrial ATF5 protein expression was decreased in the epox and CDDP groups (Figure 2e). Fiorese et al. showed that nuclear ATF5 promoted OXPHOS during mitochondrial dysfunction by inducing the expression of mitochondrial chaperones and protease genes, suggesting that nuclear ATF5 promotes cell survival by upregulating the UPR^mt^ [21]. Therefore, we speculated that epox alone initiated the UPR^mt^ to inhibit mitochondrial dysfunction-induced apoptosis to a certain extent, while the expression of UPR^mt^-related genes decreased when epox was used in combination with CDDP (Figure 2g–i). p53 activity is required for cisplatin-induced apoptosis [50]. Our results further support that epox inhibits the proteasome pathway and UPR^mt^ through p53 to increase ovarian cancer cell CDDP sensitivity.

A previous study showed that p53 overexpression in prostate cancer cells induces mitochondrial dysfunction by downregulating the expression of nuclear genes, such as *NRF1*, *TFAM*, and *SDHA* [51]. To further explore how p53 accumulated by inhibiting the regulated UPR^mt^, we investigated the expression of genes associated with the mitochondrial respiratory chain, which are co-encoded by the nuclear and mitochondrial DNA. The mRNA levels of *TFAM* and *POLRMT* decreased compared with those of the CDDP group, but only the levels of POLRMT decreased both at mRNA and the protein level, indicating that POLRMT is involved in the accumulation of p53 by inhibiting the proteasome pathway (Figure 3a,f). We measured the mRNA levels of the 13 mitochondrial respiratory chain subunits encoded by the mtDNA. The mRNA levels of most of the 13 subunits were decreased in the combined CDDP and epox group (Figure 3b–e). Furthermore, we found that wild-type p53 overexpression decreased the mRNA levels of most of the above subunits (Figure 4a–e). POLRMT has been reported to be responsible for catalyzing the transcription of the mitochondrial genome that encodes the essential 13 mtDNA-encoded OXPHOS protein subunits [52]. Therefore, under treatment with both CDDP and epox, we speculated that mitochondrial p53 accumulation caused by inhibiting the proteasome pathway decreased the POLRMT function and inhibited the UPR^mt^ by decreasing the expression of the 13 mtDNA-encoded mitochondria respiratory chain subunits(Figure 4a–e).

The shuttle protein p62 alters protein localization by transporting proteins in cells. Myeku et al. found that proteasome inhibitors led to p62 accumulation in cells [53]. We found that epox treatment in ovarian cancer cells led to p62 accumulation in the mitochondria and nucleus(Figure 5 and Figure 6), and speculated that p62 might be involved in the regulation of the nuclear and mitochondrial localization of p53. Wt-p62 and a ΔUBA-p62 were transfected into A2780 cells. After treatment with the same dose of cisplatin, compared with wt-p62, A2780 cells transfected with ΔUBA-p62 had lower protein levels of p53 in mitochondria, suggesting that p62 coordinated the localization of p53 to the mitochondria through its UBA domain (Figure 7a,b). Moreover, most of the mRNA levels of the 13 respiratory chain complex subunits encoded by mtDNA were higher in the ΔUBA-p62 group compared with those in the wt-p62 group (Figure 7c–f). Furthermore, we evaluated cell viability. As expected, deleting the UBA domain decreased the sensitivity of A2780 cells to cisplatin (Figure 7g). Our previous study showed that p62 is a bridge for Caspase 8 recruitment and activation on the autophagosome membrane in ovarian cancer cells [6]. Furthermore, mutations in the UBA domain of p62 increased the mitochondrial localization of HK2 and promoted tumor cell survival [54]. This study verified our hypothesis that p62 promotes the mitochondrial localization of p53 through its UBA domain and that it participates in regulating the sensitivity of ovarian cancer cells to cisplatin. 

**Figure 8:**
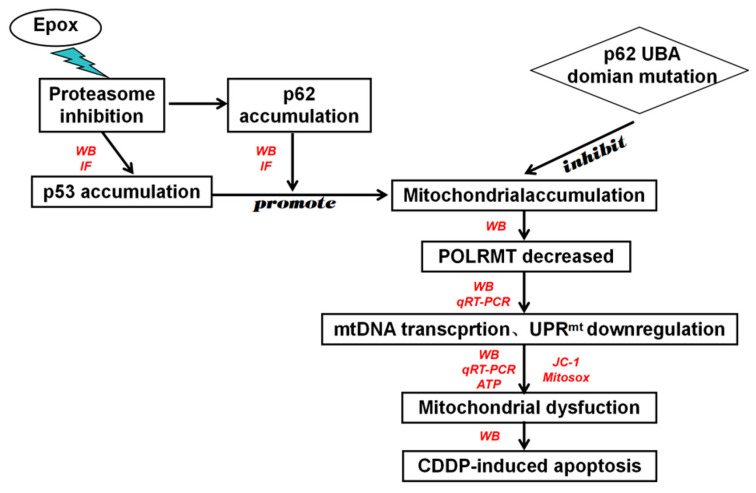
p62 promotes the mitochondrial localization of p53 through its UBA domain and participates in regulating the sensitivity of ovarian cancer cell to cisplatin. After the combined use of epox and CDDP, p62 and p53 accumulate in the mitochondria, leading to cell death by downregulating of mitochondrial DNA transcription, UPR^mt^, mitochondrial dysfunction and decreased sensitivity of A2780 cells to cisplatin, which was mediated by the UBA domain of p62. The green lightning symbol represents the use of the drug, the red text represents the detection method, the rectangle represents the experimental result, the circle represents the drug treatment, and the rhombus represents the transfection of p62 lacking the UBA domain.

In conclusion, our study provides evidence that inhibition of the proteasome pathway altered the mitochondrial localization of p53, downregulated the gene expression of mitochondrial respiratory chain subunits to impair mitochondrial functions, promoted the sensitivity of ovarian cancer cells to cisplatin, and promoted mitochondrion-dependent apoptosis, which was coordinated by the UBA domain of p62 (Figure 8). These data contribute to understanding the role of proteasome inhibitors that target p53 protein homeostasis in cancer therapy. By exploring the interaction between p53 and p62, a comprehensive understanding of the complexity of mitochondrial–nuclear communication that co-regulates mitochondria will provide a basis for the development of new targeted therapies for cancer.

## 4. Materials and Methods

### 4.1. Cell Culture

The human ovarian cancer cell lines A2780 and SKOV3 were purchased from the Shanghai Cell Bank of Chinese Academy of Sciences (Shanghai, China). Both cells lines were cultured in RPMI 1640 (Gibco Life Technologies, Carlsbad, CA, USA),supplemented with 10% fetal bovine serum(Invitrogen, Carlsbad, CA, USA), 100 IU/mL streptomycin and 100 IU/mL penicillin, at 37 °C under humidified air containing 5% CO_2_.

### 4.2. Reagents and Antibodies 

Cisplatin and epoxomicin were purchased from MedChemExpress (Monmouth Junction, NJ, USA). 3-(4, 5-Dimethylthiazol-2-yl)-2, 5-diphenyltetrazolium bromide (MTT) were purchased from Sigma-Aldrich (St. Louis, MO, USA). The following antibodies were used: anti-p62, anti-Mcl-1 (Abcam, Cambridge, MA, USA); anti-p53,anti-β-actin, anti-VDAC1, anti-LaminA/C, anti-βtublin, anti-p53, antiBax (Proteintech group, Inc., Rosemont, IL, USA); anti-PORLMT (Cell Signaling Technology, Danvers, MA, USA); anti-Bcl-2 (PTI BIO, Liaoning, China); anti-OXPHOS (Thermo Fisher Scientific, Waltham, MA, USA); All antibodies were diluted at a ratio of 1:1000.

### 4.3. Cell Viability Assays

Cells were seeded in 96-well plates at a density of 8 × 103 cells per well (A2780) or 10 × 103 cells per well (SKOV3) overnight. The cells were first treated with different reagents for the indicated times and then treated with the MTT reagents. After 4–6 h, we measured the absorbance at 490 nm using a Multiskan Spectrum (BioTek, Winooski, VT, USA).

### 4.4. Mitochondrial Membrane Potential (MMP) Detection

A2780 cells were seeded at a density of 4 × 105 cells per well in a 6-well plate, treated with cisplatin or epox or both for 12 h, and then stained using a Mitochondrial Membrane Potential Assay Kit with JC-1 (Beyotime, Beijing, China), following the manufacturer’s instructions. Briefly, cells were incubated with JC-1 dye at 37 °C under humidified air containing 5% CO_2_ for 20 min in the dark. Then, the cells were washed with staining buffer and centrifuged at 600× *g* for 4 min at 4 °C thrice. Finally, cells were suspended in staining buffer and analyzed using a BD Accuri C6 flow cytometer (BD Biosciences, CA, USA).

### 4.5. ATP Measurement

ATP measurement was conducted using an Enhanced ATP Assay Kit (Beyotime, Beijing, China). Briefly, cells were lysed with a lysis buffer and centrifuged at 4 °C (10,000× *g*, 5 min). The ATP level was determined by mixing 10 μL of the supernatant with 10 μL of luciferase reagent using a luminometer (BMGLabtech, Ortenberg, Germany) RLU value or CPM.

### 4.6. Detection of the Mitochondrial ROS Levels

The mitochondrial ROS levels were measured using mitosox (Thermo Fisher Scientific, MA, USA) following the manufacturer’s instructions. Briefly, cells were stained with mitosox dye, incubated at 37 °C for 10 min, and washed thrice with cold PBS. The samples were analyzed using Accuri C6 Flow Cytometry (BD Biosciences, FranklinLakes, NJ, USA).

### 4.7. Determination of the Relative mtDNA Copy Number

Total DNA was extracted using a TIANamp Genomic DNA kit (Tiangen Biotech Co., Ltd., Beijing, China). qPCR was used to measure the mitochondrial-encoded nicotinamide adenine dinucleotide dehydrogenase 1 (ND1) level relative to that of nuclear-encoded gene 18S rRNA (18S).

The following primer sequences were used:

18srRNA:5′-TAGAGGGACAAGTGGCGTTC-3′ (forward) and

5′-CGCTGAGCCAGTCAGTGT-3′ (reverse).

ND1:5′-CACCCAAGAACAGGGTTTGT-3′ (forward) and

5′-TGGCCATGGATTGTTGTTAA-3′ (reverse).

### 4.8. Western Blot Analysis

Cells were lysed using RIPA lysis buffer containing proteasome inhibitors. Lysates were centrifuged at 4 °C and 4500 rpm for 10 min, boiled in loading buffer and resolved using SDS-PAGE. Proteins were transferred to PVDF membranes, which were later blocked with 5% skim milk and then incubated with primary antibodies overnight at 4 °C. The next day, the membranes were incubated with horse radish peroxidase (HRP)-conjugated secondary antibodies (Proteintech, Chicago, IL, USA) according to the manufacturer’s instructions. The ECL reagent (Thermo Fisher Scientific, MA, USA) was used for immune-detection and band visualization using Syngene Bio Imaging (Synoptics, Cambridge, UK). β-actin was used as an endogenous control, VDAC as a mitochondrial control, and LaminA/C as a nuclear control.

### 4.9. Cell Transfection

The pcDNA3.1 vector (NC), pcDNA3.1-p62(wt-p62), pcDNA3.1-ΔLIR-p62, and pcDNA3.1-ΔUBA-p62 were purchased from Sangon Biotech (Shanghai, China). Cells were seeded in 6-well plates overnight and then transiently transfected with pcDNA-plasmid prepared in the laboratory using the Lipofectamine Transfection Reagent (Thermo Fisher Scientific, MA, USA) according to the manufacturer’s protocols.

### 4.10. RNA Extraction and Quantitative Real-Time PCR(qPCR)

Total RNA extraction and RT-qPCR was performed as previously described before [55]. The primer sequences are presented in Table 1. The target gene mRNA levels were normalized to those of *β-actin* (Sangon Biotech, Shanghai, China). Primers sequences are presented in Table 1.

### 4.11. Mitochondria Isolation

Cells were harvested after treatment with CDDP or epox or both for 0, 6, and 12 h. Mitochondria extraction was conducted using a cell mitochondria isolation kit (Beyotime, Beijing, China) following the manufacturer’s instructions. Briefly, cells were washed with cold PBS, digested with trypsin-EDTA solution, and centrifuged at 600× *g* at 4 °C for collection. The supernatant was discarded and cells were resuspended in PMSF-supplemented mitochondrial separation reagent and placed in an ice bath for 10–15 min. The cell suspension was transferred to a glass homogenizer of appropriate size, and homogenized approximately 10–30 times. The cell homogenate was centrifuged at 600× *g* and 4 °C for 10 min, and the supernatant was transferred to another centrifuge tube and centrifuged at 11,000× *g* and 4 °C for 10 min. The supernatant was subsequently discarded. The pellet contained the isolated cell mitochondria. The supernatant, which contained the cytoplasmic protein, was centrifuged at 12,000× *g* for 10 min at 4 °C. The supernatant was obtained to isolate mitochondrial cytoplasmic proteins. 

### 4.12. Nuclear Isolation

Cells were harvested after treatment with CDDP or epox or both for 12 h. Nuclear protein extraction was conducted using a Nuclear and Cytoplasmic Protein Extraction Kit (Beyotime, Beijing, China) following the manufacturer’s instructions. Briefly, cells were washed with cold PBS, scraped with cell scrapers, centrifuged, and collected. The supernatant was discarded, and PMSF-supplemented cytoplasmic protein extraction reagent A was added to the cell pellet, followed by vortexing at maximum speed for 5 s. The sample was subsequently placed in an ice bath for 10–15 min. Cytoplasmic Protein Extraction Reagent B was then added, and the sample was vortexed at top speed for 5 s, and placed in an ice bath for 1 min, followed by vortexing at top speed for 5 s, and centrifugation at 12,000–16,000× *g* for 5 min at 4 °C. The supernatant contained the extracted cytoplasmic protein. PMSF-supplemented nuclear protein extraction reagent was added to the cell pellet and the sample was vortexed at maximum speed for 15–30 s, then placed back in the ice bath, and vortexed at maximum speed for 15–30 s every 1–2 min for a total of 30 min. Next, samples were centrifuged at 12,000–16,000× *g* for 10 min at 4 °C. The supernatant contained the extracted nuclear protein. 

### 4.13. Immunofluorescence

The cells were seeded in 24-well plates and treated with drugs at a cell confluence of 70–80%. Cells were stained with mitotracker red(Invitrogen, Carlsbad, CA, USA) for 20 min in the dark at 37 °C, and then, washed thrice with PBS and fixed with 4% paraformaldehyde for 25 min. Next, cells were washed with PBS and permeabilized with 0.1% Triton-PBS for 7 min. After being washed with PBS and blocked with 5% horse serum for 30 min, cells were incubated with primary antibodies overnight at 4 °C. The next day, cells were washed with PBS and incubated with with FITC/Texas Red–conjugated secondary antibodies (Proteintech, Chicago, IL, USA) for 30 min at room temperature in the dark. The cells were washed with PBS, stained with Hochest33342 for 5 min. The images were acquired using an Echo Lab Revolve microscope (San Diego, CA, USA).

### 4.14. Statistical Analysis

Statistical analysis was performed using GraphPad Prism 8 (La Jolla, CA, USA). All of the data are presented as means ± standard deviation (SD). All experiments were repeated three times. A one-way ANOVA followed by Student’s *t*-test and Tukey’s tests were used to compare differences among groups.

## Figures and Tables

**Table 1:** Primer Sequences.

Primer Name	Sequence (5′-3′)	Primer Name	Sequence (5′-3′)
*MT-ND1*	ATGGCCAACCTCCTACTCCTCATT TTATGGCGTCAGCGAAGGGTTGTA	*MT-ATP6*	TAGCCCACTTCTTACCACAAGGCA TGAGTAGGTGGCCTGCAGTAATGT
*MT-ND2*	ACTGCGCTAAGCTCGCACTGATTT GATTATGGATGCGGTTGCTTGCGT	*MT-ATP8*	ACCGTATGGCCCACCATAATTACC TTTATGGGCTTTGGTGAGGGAGGT
*MT-ND3*	CCCTACCATGAGCCCTACAAACAA AGTCACTCATAGGCCAGACTTAGG	*TFAM*	ATGGCGTTTCTCCGAAGCAT TCCGCCCTATAAGCATCTTGA
*MT-ND4*	ACAAGCTCCATCTGCCTACGACAA TTATGAGAATGACTGCGCCGGTGA	*TFB2M*	GACCACTTACGTTCATTGACTCC CAGGGTTTCATCATACAGCCAT
*MT-ND4L*	TATCGCTCACACCTCATATCCTCCCT AGGCGGCAAAGACTAGTATGGCAA	*POLRMT*	GCAAGACCAAGACCGCAGGAAG GCTACCATCTCCACTGCCACATTC
*MT-ND5*	ATCGGTTTCATCCTCGCCTTAGCA ACCTAATTGGGCTGATTTGCCTGC	*mtHSP70*	CAAGCGACAGGCTGTCACCAAC CAACCCAGGCATCACCATTGG
*MT-ND6*	AGGATTGGTGCTGTGGGTGAAAGA ATAGGATCCTCCCGAATCAACCCT	*HSP60*	GATGCTGTGGCCGTTACAATG GTCAATTGACTTTGCAACAGTCACAC
*MT-CO1*	ACCCTAGACCAAACCTACGCCAAA TAGGCCGAGAAAGTGTTGTGGGAA	*HSP10*	TGGCAGGACAAGCGTTTAG GGTTACAGTTTCAGCAGCAC
*MT-CO2*	ACAGATGCAATTCCCGGACGTCTA GGCATGAAACTGTGGTTTGCTCCA	*Lonp1*	AGCCTTATGTCGGCGTCTTTC CGTCCCCGTGTGGTAGATTTC
*MT-CO3*	ACTTCCACTCCATAACGCTCCTCA TGGCCTTGGTATGTGCTTTCTCGT	*p53*	CAGCACATGACGGAGGTTGT TCATCCAAATACTCCACACGC
*MT-CYB*	TCCTCCCGTGAGGCCAAATATCAT AAAGAATCGTGTGAGGGTGGGACT		

## Data Availability

Not applicable.
